# Reliable Analytical Approach for the Quantification of the Cholesterol Adsorption Capacity of Artichoke Leaves

**DOI:** 10.3390/foods15173099

**Published:** 2026-08-31

**Authors:** Shahd Ali, Federica Ianni, Lina Cossignani

**Affiliations:** Department of Pharmaceutical Sciences, University of Perugia, Via S. Costanzo, 06126 Perugia, Italy; shahd.ali@dottorandi.unipg.it (S.A.); lina.cossignani@unipg.it (L.C.)

**Keywords:** plant matrices, artichoke leaves, dietary fibers, cholesterol adsorption capacity, chromatography, spectrophotometry

## Abstract

Reliable determination of cholesterol adsorption capacity (CAC) is essential for evaluating the hypocholesterolemic potential of dietary fibers; however, currently available analytical procedures lack methodological standardization and often rely on spectrophotometric assays susceptible to matrix interference. The present study aimed to develop and validate a simple and reliable procedure for the evaluation of CAC of fractionated artichoke leaves. In vitro assays were conducted under simulated gastrointestinal conditions, with residual cholesterol quantified using high-performance liquid chromatography and two different detection systems (UV-Vis spectrophotometric detector and light-scattering detector). The chromatographic method was validated in terms of linearity, sensitivity, precision, and accuracy, and its performance was compared with that of the conventional ortho-phthalaldehyde (OPA) spectrophotometric assay. Some pure commercial fibers were tested first; pectin and lignin showed the highest cholesterol adsorption capacity. Moreover, the results showed that all the investigated leaf fractions exhibited CAC, with the outer leaf fractions showing significantly higher values than the inner woody fractions. The proposed workflow provides a reliable and consistent analytical framework for CAC determination. Furthermore, the results provide new insights into the potential cholesterol-lowering properties of dietary fiber from artichoke leaves, supporting their further investigation as functional ingredients.

## 1. Introduction

Cardiovascular diseases (CVD) remain the leading cause of mortality worldwide, with hypercholesterolemia identified as a major modifiable risk factor. Elevated serum cholesterol, particularly low-density lipoprotein cholesterol (LDL-C), promotes atherosclerosis and vascular dysfunction [1]. While statins and other lipid-lowering drugs are effective, they may induce side effects, prompting growing interest in functional food-based strategies, particularly dietary fibers, for cholesterol management [2].

Dietary fiber exerts hypocholesterolemic effects through several mechanisms, including direct binding of cholesterol and bile acids, increasing intestinal viscosity, promoting fecal sterol excretion, and supporting microbial fermentation to short-chain fatty acids that suppress hepatic cholesterol synthesis [3]. Both soluble and insoluble fiber contribute to these effects, making plant-derived fiber an important focus of research. Nowadays, fibers from various conventional and uncommon sources, including agro-food processing by-products, are being tested. Several studies have investigated isolated or modified fibers to understand their cholesterol-lowering potential. Soluble dietary fiber from soy hulls, mainly represented by pectic polysaccharides, showed good cholesterol and bile acid binding capacity and reduced serum cholesterol in vivo [4]. Wu and colleagues investigated the cholesterol adsorption and prebiotic potential of soluble dietary fiber (SDF) and insoluble dietary fiber (IDF) obtained from different varieties of bamboo shoots [5]. They found that, generally, IDF showed better adsorption capacity than the corresponding SDF from the same bamboo shoot species. Insoluble ginseng residue fiber also showed high cholesterol adsorption capacity, equal to 49.69 mg/g at pH 7.0 [6], while a lower value (5.19 mg/g) was reported for foxtail millet bran fractions [7].

In a previous study [8], SDF from defatted rice bran resulted in a greater reduction in cholesterol concentration than IDF at pH 2 and pH 7. Wang and colleagues [9] evaluated several properties of *Ganoderma lucidum* dietary fiber, demonstrating a significantly greater cholesterol adsorption capacity for SDF (29.8%) compared to IDF under identical experimental conditions. These findings highlight that cholesterol adsorption capacity can vary substantially among dietary fibers from different sources and may depend on several factors, such as pH, viscosity, and crystalline structure, which may influence their physicochemical properties.

Recent studies have had the objective of modifying the properties of dietary fiber by specific treatments to improve its functional properties. For example, Guan and colleagues reported that fermentation increased the level of soluble dietary fiber of black rice and its adsorption capacity for water, oil, cholesterol, glucose, and sodium cholate [10]. Other authors studied the effect of different treatments on the physicochemical properties, morphological structures, and adsorption capacities in vitro of fiber purified from tea seed [11] and highlighted that the ultrasound treatment increased the adsorption properties of tea seed dietary fiber, together with its physiological properties. Although to a lesser extent, the evaluation of lipid-lowering activity of whole plant materials or minimally processed by-products has gained attention for its practicality and sustainability. For example, okra pods were subjected to superfine grinding, and their physicochemical and functional properties were studied, revealing that with decreasing okra powder size, the specific surface area, water-holding capacity, and oil and cholesterol binding capacity were increased significantly [12]. Moreover, red raspberry pomace and its total dietary fiber were investigated, aiming to evaluate their potential to be used as functional food ingredients, revealing that the first had significantly higher in vitro cholesterol and glucose adsorption capacities [13]. These findings highlight the potential of valorizing agro-industrial residues as functional food components without complex fractionation.

In this context, the availability of a reliable and consistent procedure for measuring cholesterol adsorption capacity is of great relevance. Cholesterol can be determined by several analytical methods, such as gravimetry, colorimetry, fluorimetry, and chromatography [14]. Enzymatic assays coupled with spectrophotometric detection, while well suited for clinical serum analyses, prove less reliable in food matrices due to interfering lipids and incomplete extraction [15]. Chromatographic techniques, especially reversed-phase liquid chromatography and gas chromatography, have emerged as the gold standard for sterol analysis, offering enhanced selectivity by efficiently resolving cholesterol from other sterols and oxidation products [16,17,18]. However, the robustness of these chromatographic methods remains highly dependent on the sample preparation phase, particularly the efficient lipid extraction and saponification steps [14]. Nevertheless, conventional spectrophotometry based on the ortho-phthalaldehyde (OPA) reagent is widely used for assessing the cholesterol adsorption capacity of plant materials and dietary fibers due to its simplicity, even if it may suffer from matrix interference and limited specificity. Moreover, the conditions adopted for cholesterol adsorption and sample treatment before instrumental analysis are not uniform across the various studies present in the literature.

Accordingly, the present study aims to develop a reliable procedure for testing the CAC in plant materials. To this aim, artichoke leaves were selected as they represent a largely underutilized agro-industrial by-product. In contrast to other plant-derived residues, artichoke leaves have received comparatively limited attention as a source of lignocellulosic fibers. Furthermore, leaves have also received considerably less attention than other artichoke residues, such as bracts and stems, which are generally the most investigated fractions [19,20]. For this reason, leaf valorization is of interest from both a waste-minimization and circular-bioeconomy perspective. In more detail, artichoke leaves have been selected as a case study for assessing CAC determination, with the aim of providing a relevant and reliable protocol that could potentially be extended to other plant matrices, thereby filling the existing methodological gap. In this context, HPLC coupled with light scattering detection (LSD) was assessed as a valuable alternative to the more commonly used UV-Vis detection, potentially improving selectivity and expanding the analytical possibilities for cholesterol determination in complex plant-derived matrices.

The specific objectives of this study were: (i) using a standardized in vitro digestion model to simulate salivary, gastric, and intestinal phases during the adsorption process; (ii) quantifying cholesterol levels using HPLC-UV-Vis coupled with light scattering detection (HPLC-LSD) for reliable measurement; (iii) testing CAC in standard polysaccharides and samples from artichoke leaves by the developed procedure and (iv) comparing the chromatographic results with those obtained by spectrophotometric method. This study therefore proposes a comprehensive and reliable analytical framework for evaluating CAC in plant tissues or isolated fractions, combining a well-defined in vitro digestion protocol with validated chromatographic quantification and a cross-method comparison.

## 2. Materials and Methods

### 2.1. Materials and Reagents

Cellulose fibers (C6288), microcrystalline cellulose (310697), inulin (I2255), lignin alkali (471003), and pectin (93854) were purchased from Merck (Darmstadt, Germany). Their basic structural unit and characteristics are reported in Table 1.

Cholesterol (C8667, ≥99%), o-phthaldialdehyde (OPA, P0657), α-amylase (A3403-500 KU from Bacillus licheniformis), pepsin (P7000 from Porcine Gastric Mucosa), pancreatin (P1750, from Porcine Pancreas), and bile salts (B8631) were purchased from Merck. Reagents and HPLC-grade solvents were obtained from Carlo Erba Reagents (Milan, Italy). Water was purified using a Milli-Q Plus 185 system (Millipore, Burlington, MA, USA). Fresh eggs were purchased from a local supermarket in Perugia, Italy.

### 2.2. Plant Material

Artichoke leaves (*Cynara cardunculus* subsp. *Scolymus*, “Romanesco” cultivar) were collected in May 2024 from a local farm near Perugia, Italy (altitude 368 m a.s.l., coordinates 42°55′36″ N 12°14′28″ E). The leaves were washed, dried in a forced-air oven (Binder FD53, Tuttlingen, Germany) at 40 °C for 4–5 days, and manually separated into outer (leafy) and inner (woody) portions. Each fraction was ground (Oster 869-50R, Milwaukee, WI, USA) and sieved. As indicated in a recent study [21], four samples were obtained (Table 2): A and B from outer leaves (>1.18 mm and <1.18 mm, respectively), and C and D from inner leaves (<1.18 mm and >1.18 mm, respectively). The yield of each fraction after drying, grinding, and sieving was as follows: 33.8% for fraction A, 31.8% for fraction B, 19.2% for fraction C, and 15.2% for fraction D.

### 2.3. In Vitro Digestion

The cholesterol adsorption capacity of artichoke leaf fibers and reference dietary fibers was evaluated using a static in vitro digestion model adapted from the INFOGEST 2019 protocol [22] with minor modifications to accommodate cholesterol extraction. All experiments were conducted in triplicate.

A day before digestion the reagents were prepared in 50 mL volumetric flask with the following amount of weight and molarity, KCl (1.99 g/50 mL, 0.5 M), NaH_2_PO_4_ (3.4 g/50 mL, 0.5 M), NaHCO_3_ (4.2 g/50 mL, 1 M), NaCl (5.85 g/50 mL, 2 M), MgCl_2_·6H_2_O (1.53 g/50 mL, 0.15 M), (NH_4_)_2_CO_3_ (2.4 g/50 mL, 1 M), CaCl_2_ (2.2 g/50 mL, 0.3 M) in distilled water. The acidic/basic solutions were prepared as follows: 10 mL of 37% HCl (6 M) was mixed with 10 mL of distilled water, and 1 g of NaOH (1 M) was mixed with 25 mL of distilled water. On the day of the procedure, the dried and ground artichoke leaf samples (A, B, C, D) and reference fibers (microcrystalline cellulose, fibrous cellulose, inulin, lignin, and pectin) were weighed (0.5 g) into 50 mL polypropylene tubes. Then, 1 g of fresh egg yolk, manually separated from the albumen and homogenized, was added.

Samples were hydrated with 2 mL of preheated simulated salivary fluid (SSF) and manually mixed for 1 min. 12.5 µL of CaCl_2_ and 0.75 mL of α-amylase solution (75 U/mL per sample) were added, and the total volume was adjusted to 5 mL with distilled water. Samples were incubated at 37 °C with continuous shaking at 100 rpm for 2 min to simulate oral digestion, using the shaking incubator (BSI-2/SKI 4, Suzhou Being Medical Device Co., Ltd., Suzhou, China).

After the oral phase, 4 mL of simulated gastric fluid (SGF) was added, mixed manually for 1 min, followed by the addition of 2.5 µL CaCl_2_ and 0.5 mL pepsin solution (2000 U/mL). The pH was adjusted to 3.0 using HCl (6 M), and the volume was brought to 10 mL with distilled water. The pH was measured with a digital pH meter (Model 334-B, AMEL S.r.l., Milan, Italy). Samples were incubated at 37 °C and 100 rpm for 2 h.

Before intestinal digestion, enzyme solutions were prepared: pancreatin (100 U/mL) and bile salts (0.1 M in simulated intestinal fluid, SIF). To each gastric digest, 4.25 mL SIF, 2.5 mL pancreatin, 1.25 mL bile salts, and 20 µL of CaCl_2_ (0.3 M) were added. The pH was adjusted to 7.0 using NaOH (1 M), and the final volume was adjusted to 20 mL with distilled water. Samples were incubated at 37 °C and 100 rpm for 2 h.

Post-digestion, samples were centrifuged at 4000 rpm for 30 min (Neya 8, Remi Elektrotechnik Ltd., Mumbai, India). Supernatants were collected and stored at 4 °C overnight. The following day, samples were re-centrifuged under the same conditions to ensure clarity before alkaline hydrolysis and cholesterol extraction.

### 2.4. Sample Preparation for Cholesterol Instrumental Analysis

Lipid residues were saponified according to [23], with some modifications: an aliquot of 5 mL of supernatant was combined with 3 mL of ethanol and 2 mL of 50% *w*/*v* KOH in new falcons with magnetic stirring, mixed, and incubated in a 60 °C water bath with stirring for 1 h. Samples were cooled in an ice bath, then 5 mL of hexane and 3 mL of distilled water were added. Mixtures were vortexed for 1 min (ZX3, VELP Scientifica Srl, Usmate, Italy), repeated for 3 turns each, and centrifuged at 5000 rpm for 20 min, twice. 2 mL of the upper hexane layer was collected and evaporated under nitrogen gas at room temperature. The dried residue was reconstituted in 2 mL of the same phase used as the HPLC mobile phase, acetonitrile/isopropanol (70:30 *v*/*v*), sonicated, and vortexed before injection.

### 2.5. HPLC Analysis of Cholesterol

High-performance liquid chromatography (HPLC) analysis was carried out on a Shimadzu SPD-10A VP system (Kyoto, Japan) coupled to a UV-Vis detector from the same manufacturer and a light-scattering detector (SEDEX 55, S.E.D.E.RE., Alfortville, France), and equipped with a Rheodyne 7725i injector (Rheodyne Inc., Cotati, CA, USA) and a 20 μL stainless steel loop. Signal acquisition and data handling were performed with Chrom&Spec software (Chromatographic Specialties Inc., Brockville, ON, Canada). For both detection systems, a Robusta^®^ 100A C18 column (250 × 4.6 mm, 5 µm; Sepachrom, Milan, Italy) was used with a mobile phase consisting of a mixture of acetonitrile/isopropanol (70:30, *v*/*v*). The injection volume was 20 µL, and the column temperature was maintained at 30 °C.

Cholesterol identification with both detection systems was performed by comparing the retention time of the analyte in the samples with that of the cholesterol standard. The identity of the cholesterol peak was further confirmed by spiking the samples with the authentic standard and verifying the corresponding increase in peak response without any change in retention time.

With a UV-Vis detector, cholesterol was monitored at 210 nm. For quantification, standard curves were prepared using cholesterol solutions in the same mobile phase over the range 0.00175–0.75 mg/mL.

LSD system was set at a drift tube temperature of 80 °C and a nitrogen pressure of 240 kPa. Cholesterol quantification was performed using calibration standards in the range 0.00175–1.0 mg/mL.

### 2.6. HPLC Analysis Validation

The analytical procedures for cholesterol quantification by HPLC/UV and HPLC/LSD were validated following the International Council for Harmonization (ICH) guideline Q2(R2) [24]. Validation parameters included linearity, limit of detection (LOD), limit of quantification (LOQ), precision (intra- and inter-day), accuracy (recovery), and selectivity.

Linearity was evaluated using cholesterol standard solutions prepared in the mobile phase at multiple concentration levels covering low and high ranges.

For both systems, separate calibration curves were constructed for low and high concentrations. For HPLC-LSD, logarithmic transformation of the peak area was applied to achieve linearity across the tested range. Calibration curves were obtained by plotting peak area (or log-transformed area for LSD) versus concentration, and linear regression analysis provided the slope (S) and correlation coefficients (R^2^).

The evaluated linearity ranges for the HPLC-UV system were 1.75–73 μg/mL at the low level (Low-Level validation, LLV) and 146–750 μg/mL at the high level (High-Level validation, HLV). For the HPLC-LSD system, the corresponding linearity ranges were 3.5–146 μg/mL (LLV) and 146–750 μg/mL (HLV).

LOD (Limit of Detection) and LOQ (Limit of Quantification) are crucial analytical parameters used to define the lowest level that can be reliably detected (LOD, signal-to-noise ratio of ~3.3) and accurately quantified (LOQ, signal-to-noise ratio of ~10). A commonly accepted procedure for estimating LOD and LOQ is based on independent measurements of a blank sample. The standard deviation (SD) of these measurements is then calculated, which corresponds to the standard deviation of the instrumental noise.

In line with this approach, ten blank injections were run using both detection systems to establish the background noise expressed as the limit of blank (LOB), which is essential for calculating the two above parameters [25]. The method assumes that if analyte is present, it will produce a signal greater than the analytical noise in the absence of analyte, according to the following equations [24,26]:

(1) LOD = Mean blank + 3.3 × SD_blank_

(2) LOQ = Mean blank + 10 × SD_blank_

The precision of the method was assessed by analyzing two external cholesterol standard solutions at low and high concentrations within a single day (intra-day, *n* = 3) and across three consecutive days (inter-day, n = 9). Precision was expressed as relative standard deviation (%RSD) among the concentration values achieved from consecutive injections:

(3) RSD (%) = SD of measured concentrations/mean × 100

Accuracy was evaluated through recovery experiments. The established external test solutions corresponding to the theoretical concentrations at the low and high levels were analyzed with both detection systems and analyzed in triplicate. The intra-day and inter-day recovery was estimated, and recovery (%) was calculated as:

(4) Recovery (%) = measured concentration/theoretical concentration × 100

All validation experiments were conducted under the chromatographic conditions described in Section 2.5.

This approach ensured that both HPLC/UV and HPLC/LSD methods were adequately validated for linearity, precision, accuracy, and sensitivity, consistent with ICH Q2(R2) [24] acceptance criteria.

### 2.7. Spectrophotometric Analysis of Cholesterol

Cholesterol derivatization with o-phthalaldehyde (OPA) reagent was performed for spectrophotometric detection according to the modified procedure of Rudel and Morris [23], following the same sample preparation as the HPLC procedure. The OPA reagent was freshly prepared before use (0.1 mg/mL in acetic acid). Briefly, 1 mL of hydrolyzed extracts was evaporated using nitrogen gas at room temperature and reconstituted in 1 mL of acetic acid (AA). 100 µL of the sample was dissolved with 200 µL of AA in a new glass tube. 2 mL of H_2_SO_4_ (96–97%) was carefully added to the inner wall of the glass tube, and 1.5 mL of OPA reagent was added; then the tube contents were vortexed immediately. The samples were incubated at room temperature for 10 min. Absorbance was measured at 550 nm using a UV–Vis spectrophotometer (Lambda 20, Perkin Elmer, Norwalk, CT, USA) against a blank prepared with the same volume of AA instead of the sample. The cholesterol concentration of unknown samples was determined against the standard curve generated by cholesterol standard solutions ranging from 0.02 to 0.05 mg/mL. All measurements were performed in triplicate.

### 2.8. Calculation of Cholesterol Adsorption Capacity (CAC)

The cholesterol adsorption capacity (CAC) was calculated after the in vitro digestion procedure based on the difference between the cholesterol content of the control (without fiber) and the cholesterol remaining in the digest containing fiber. The CAC value, expressed as milligrams of cholesterol absorbed per gram of sample (mg/g), was determined according to Equation (5):

(5) CAC (mg cholesterol/g) = (Cc − Cs)/g

where

Cc is the cholesterol amount measured for the control (after digestion without the sample), and Cs is the cholesterol amount measured for the sample (after digestion with the sample); g is the dry weight of the sample in grams.

CAC was also expressed as adsorption percentage (%), according to Equation (6):

(6) CAC (%) = (Cc − Cs)/Cc ×100

This approach quantifies the sample’s ability to bind and retain cholesterol during simulated gastrointestinal digestion, reflecting its potential for cholesterol-lowering functionality.

### 2.9. Statistical Analysis

All experiments were performed in triplicate, and results were expressed as mean ± standard deviation (SD). Statistical significance was assessed using one-way analysis of variance (ANOVA) followed by Tukey’s HSD (honestly significant difference) post hoc test. A value of *p* < 0.05 was considered statistically significant.

GraphPad PRISM version 9.3.1 (GraphPad Software, Boston, MA, USA) and Excel Version 16.91 (Microsoft, Redmond, WA, USA) for Windows were used for graph generation and statistical analysis.

## 3. Results and Discussion

### 3.1. Conditions Developed for in Vitro Testing of the Adsorption of Cholesterol

**Figure 1:**
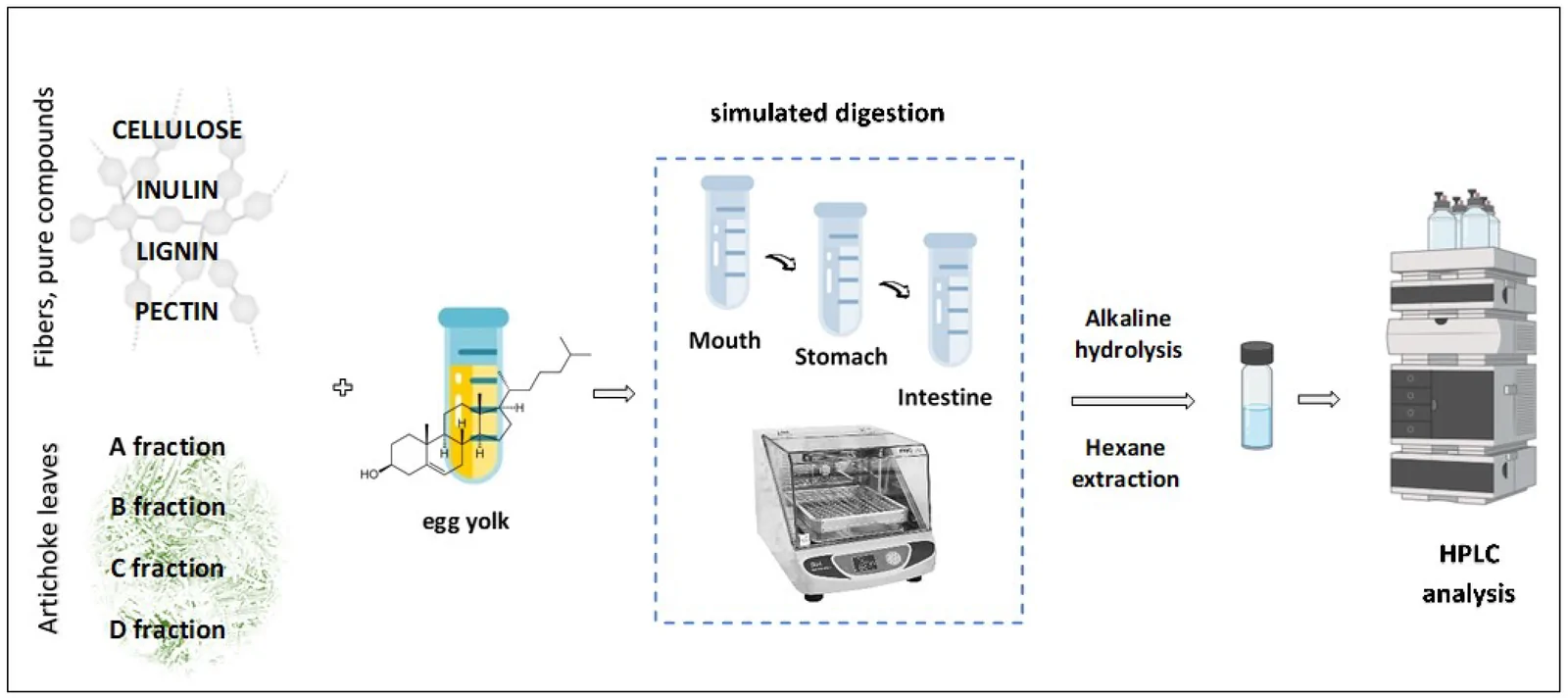
Scheme of the procedure developed for assessing the cholesterol adsorption capacity.

The primary objective of this work was to develop a reliable procedure for in vitro assessment of the cholesterol adsorption capacity of plant material. Figure 1 shows the adopted procedure. Pure fiber compounds and artichoke leaf fractions were tested using egg yolk as a cholesterol source, as it represents the most suitable model system for determining the binding capacity of cholesterol, a molecule that is difficult to dissolve in water, even after the addition of emulsifiers.

Egg yolk is widely used to test the CAC of plant materials, apart from a few exceptions, including research by Chen and coworkers, which tested okra powder by mixing it with a cholesterol solution in glacial acetic acid [12].

From an analytical perspective, cholesterol is often extracted from food matrices using mixtures of polar and non-polar solvents to improve extraction efficiency, as cholesterol may interact with other matrix components such as proteins, lipoproteins, and phospholipids [14,17].

In this study, fresh egg yolk was used as the cholesterol-containing matrix for the in vitro digestion, rather than an isolated or purified cholesterol source. This approach was considered a more representative and realistic model to mimic the conditions under which cholesterol is present in a food matrix during digestion. Therefore, potential interactions between cholesterol and other egg-yolk constituents are regarded as an inherent characteristic of the experimental model and should be taken into account when CAC results are interpreted. Importantly, matrix-matched recovery experiments were not performed; therefore, the observed reduction in cholesterol cannot be attributed exclusively to dietary fiber adsorption, as potential cholesterol losses associated with the digestion and extraction procedures cannot be completely excluded.

As the first step of the procedure, a standardized in vitro method commonly used for simulating human gastrointestinal digestion, the Infogest protocol [22], was adopted. This simple and reproducible digestion model allows researchers to study the behavior of food compounds in oral, gastric, and intestinal phases by mimicking physiological conditions (pH, enzymes, electrolytes), and it is very useful in predicting outcomes of in vivo digestion and the efficacy of functional foods. Even if static digestion methods have some limitations and cannot mimic the complex dynamics of the digestion process, their wide use is based on the relatively low cost and the easy assessment of each digestion phase, good intra- and inter-laboratory reproducibility, and robustness [22]. Although simulated digestion is nowadays pivotal to investigate the behavior of bioactive compounds in the gastrointestinal environment, unexpectedly, most studies carried out to assess the healthy properties of plant material or isolated fibers to adsorb cholesterol and inhibit its intestinal uptake are carried out by simply treating the sample at pH 2.0 and 7.0, as will be discussed in Section 3.4.

The following step in CAC assessment is the after-digestion sample treatment, a critical step for sample preparation before instrumental analysis. The procedure most adopted by researchers involves the addition of ethanol to the aqueous environment to precipitate the soluble fiber, as the only treatment before the OPA reaction and spectrophotometric analysis. The proposed sample treatment consists of alkaline hydrolysis of the sample, also named saponification, and cholesterol extraction. Lipid saponification has the main objectives of hydrolyzing acylglycerols and esters of cholesterol. The successive extraction of cholesterol into a non-polar solvent is a crucial step for the analysis of cholesterol. It has been reported that direct saponification and hexane extraction yielded sufficient recovery of cholesterol in foods, even in complex matrices such as meat and egg yolk [13]. The low polarity of hexane limits the formation of emulsions, which can impact the extraction and separation steps for cholesterol determination.

Regarding chromatographic analysis, since cholesterol possesses a single hydroxyl group that imparts slight polarity, both normal-phase (NP) and reversed-phase (RP) HPLC systems can be used for its separation. Even though NP-HPLC analysis of cholesterol has been used [27,28], RP-HPLC is preferred because it offers a wider range of column selectivity and higher performance. In the present study, a reversed-phase system based on a C18 stationary phase and a polar organic mobile phase was employed. The acetonitrile/isopropanol mixture (70:30, *v*/*v*) was chosen as the solvent system, as it provided the most stable baseline and the best chromatographic performance, yielding symmetrical, sharp peaks with a high signal-to-noise ratio and short time of analysis. Comparable reversed-phase mobile phases have been reported in the literature, including acetonitrile/isopropanol (95:5, *v*/*v*) for simultaneous cholesterol and cholesterol oxide analysis in milk [29], acetonitrile/ethanol (50:50, *v*/*v*) [30], and methanol/isopropanol (70:30, *v*/*v*) [31] for cholesterol analysis in various food matrices.

Lastly, a double-detection system, consisting of a spectrophotometer detector (UV/Vis) connected in series with a light scattering detector (LSD), was used. The choice of testing the underutilized LSD as an alternative to the most common UV or DAD detector was aimed at widening the possibilities of analysis, considering that HPLC analysis of cholesterol using UV detection at 210–215 nm faces significant selectivity problems due to the relatively low molar absorptivity of cholesterol and potential interferences from matrix components that also absorb strongly in the low-UV range [32]. For this reason, LSD was considered a valuable alternative, potentially improving selectivity and expanding the analytical possibilities for cholesterol determination in complex plant-derived matrices.

### 3.2. Validation of HPLC Analysis for Cholesterol Analysis

The analytical performance of the HPLC-UV/LSD system for cholesterol quantification in an in vitro digestion matrix was evaluated in terms of linearity, precision, accuracy, and sensitivity. Both methods exhibited excellent linearity across the tested ranges, with correlation coefficients of R^2^ ≥ 0.9893 for HPLC/UV and R^2^ ≥ 0.9853 for HPLC/LSD (Table 3). The calculated LOD and LOQ values confirmed the high sensitivity of both systems and their suitability for detecting and quantifying cholesterol after its adsorption by fiber-containing samples.

Precision was evaluated at the two tested concentrations across the range of interest. Intra-day precision, expressed as relative standard deviation (RSD%), for the two external solutions ranged, respectively, between 1.50 and 6.34% and 1.25–1.49% for the HPLC/UV system and between 3.40% and 6.44% and 0.42% and 0.93% for the HPLC/LSD system (Table 4).

Inter-day RSD% values for the two external solutions were 4.69% and 2.26% for the HPLC/UV system, and 6.71% and 1.17% for the HPLC/LSD system. Overall, the obtained results were within acceptable limits, indicating the closeness of agreement between independent results obtained under both short-term and long-term conditions.

Recovery was evaluated as the closeness of the analytical results obtained at each concentration level within the specified analytical range to the corresponding true values. Recovery values slightly above 100% were observed and can be attributed to positive bias in the analytical method rather than to actual overestimation of analyte content or enhanced method efficiency (Table 4). Interestingly, higher values were mainly observed at the lowest calibration level and were more pronounced for the UV-Vis detector than for LSD. This behavior may be related to low response specificity, in particular of the UV detector, at low wavelengths and low analyte concentrations, which is more sensitive to the contribution of co-eluting interferences and contaminants from the solvent. However, such values are generally considered acceptable when accompanied by good precision.

In conclusion, the obtained results demonstrated that the analytical protocols are fit for their intended purpose and meet the performance criteria recommended by the ICH Q2(R2) guidelines. The developed methods proved to be precise and accurate, thereby providing a reliable basis for data interpretation and ensuring reproducibility for the intended application. Furthermore, the possibility to switch between different detection systems is particularly profitable, as it increases method flexibility and transferability, reducing dependence on a single analytical platform when multicentre studies are intended. Accuracy, evaluated through recovery experiments at low and high concentration levels, ranged from 100.11 to 118.42% for intra-day measurements and from 102.59 to 114.44% for inter-day measurements, as shown in Table 4.

**Figure 2:**
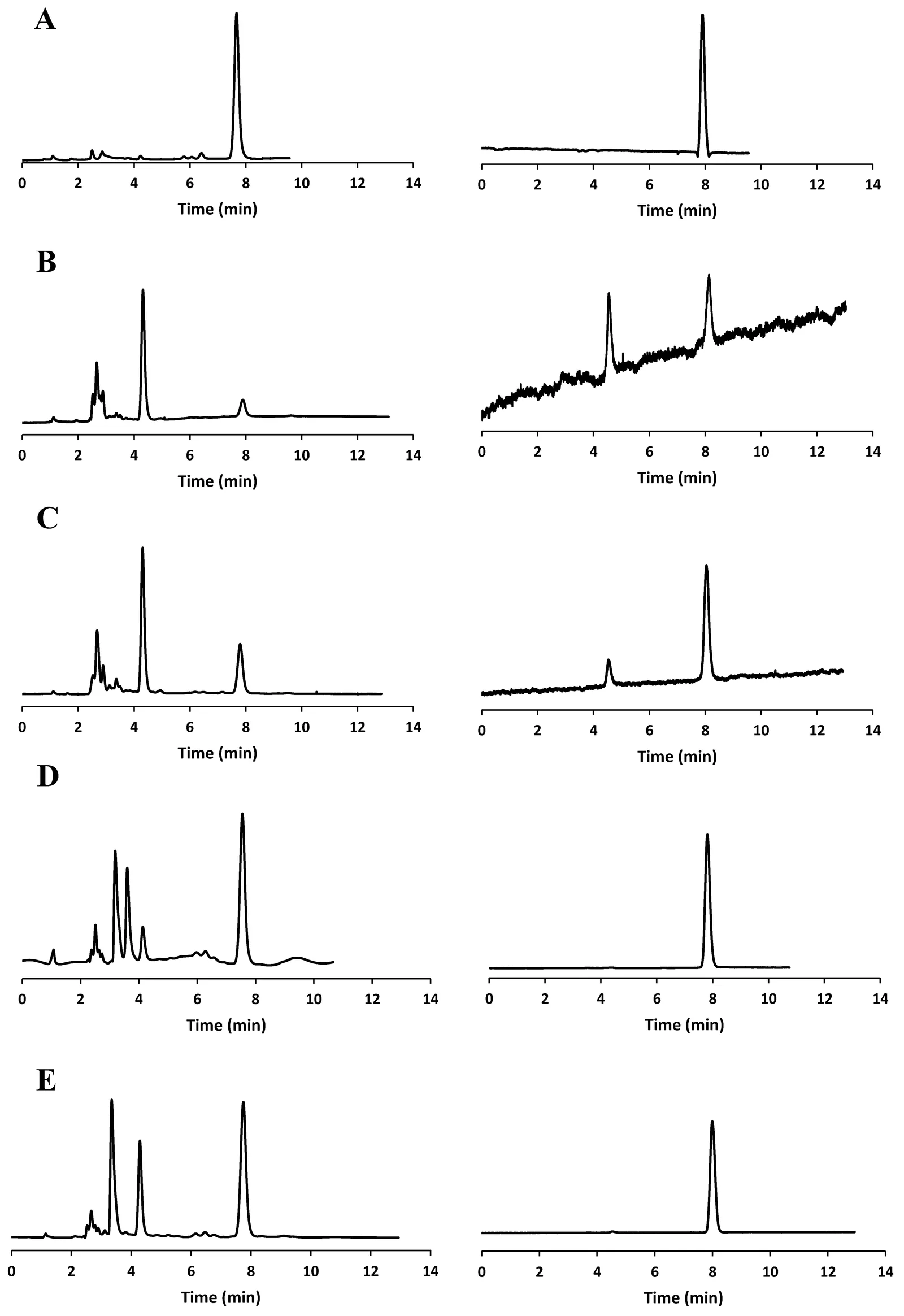
Overlay chromatograms of cholesterol standards (**A**) and cholesterol after treatment with artichoke leaf fractions (**B**–**E**) obtained by HPLC-UV-Vis (**left** column) and HPLC-LSD (**right** column). (**B**–**E**): cholesterol chromatograms after treatment with samples (**A**), (**B**), (**C**), and (**D**), respectively.

The chromatographic profiles of cholesterol after treatment with the artichoke fractions, used to determine cholesterol adsorption capacity, are shown in Figure 2.

OPA-spectrophotometry also exhibited excellent linearity (0.02–0.5 mg/mL; y = 1.011x − 0.014, R^2^ = 0.9992).

### 3.3. Results of the Cholesterol Adsorption Capacity of Pure Fibers

The developed analytical procedure was first applied to the commercial standard fibers described in the previous Table 1. The results of the CAC, expressed as mg of cholesterol per gram of sample, measured by HPLC/UV, HPLC/LSD, and by UV spectrophotometry after OPA reaction (OPA/UV), are shown in Table 5.

**Figure 3:**
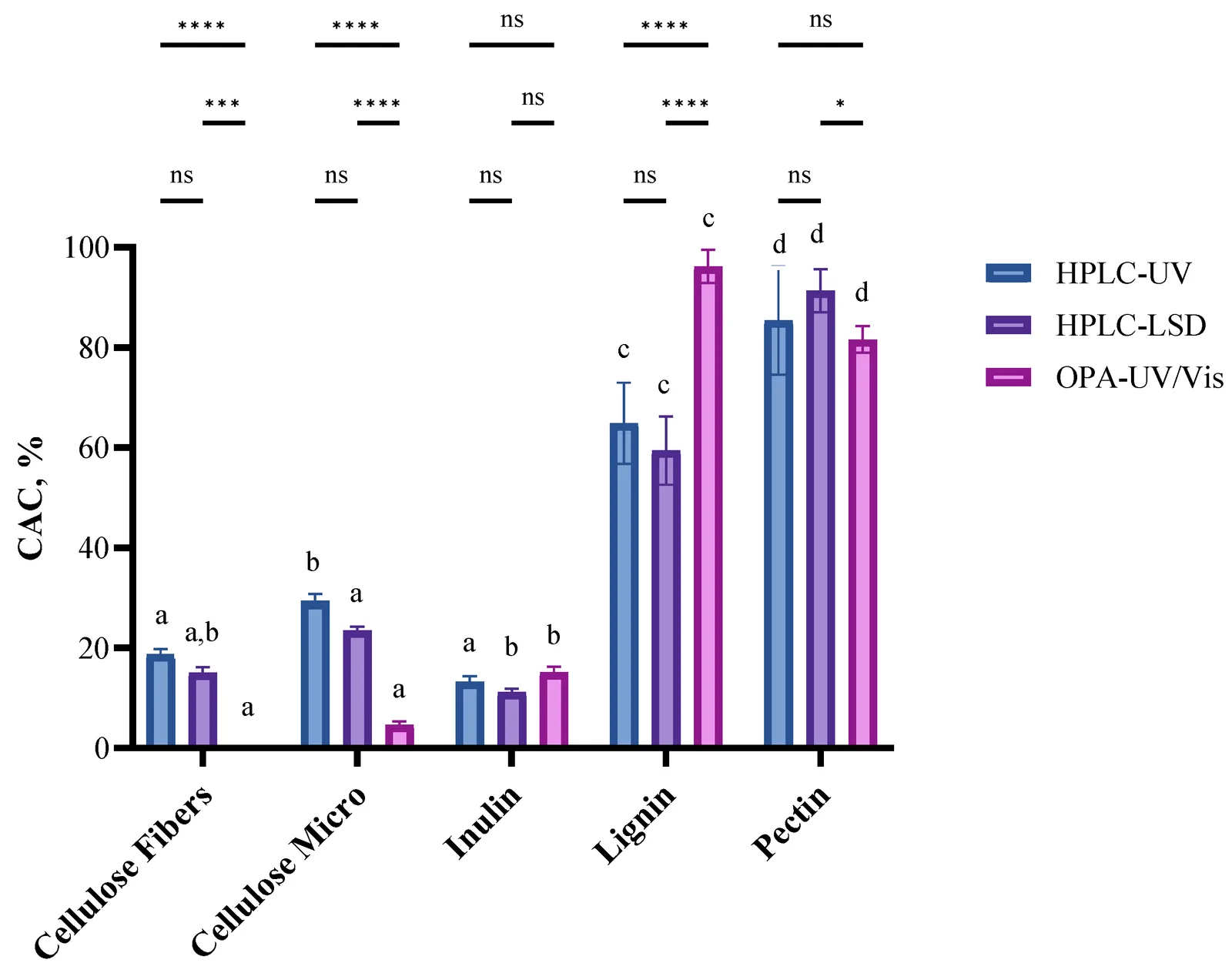
CAC of commercial fibers, expressed as adsorption percentage. Prism pairwise comparisons show differences between different analytical techniques for each compound: ns (*p* > 0.05, not significant), * (*p* ≤ 0.05), *** (*p* ≤ 0.001), and **** (*p* ≤ 0.0001). Different superscript letters above the bars indicate significantly different values (Tukey’s test, *p* ˂ 0.05) comparing the results of different compounds for each analytical technique.

From the HPLC results, it is possible to observe that pectin exhibited the highest CAC value, followed by lignin, while inulin showed the lowest. Moreover, it is interesting to highlight that HPLC results obtained with the two detection systems were not statistically different (*p* > 0.05). Regarding OPA/UV, higher results (*p* < 0.05) were observed for lignin, followed by pectin, while microcrystalline cellulose exhibited the lowest value, and the CAC of the fibrous one was even undetected. Comparison of the HPLC and spectrophotometric techniques revealed significantly higher values for the OPA/UV method in all samples, except for microcrystalline cellulose, which yielded higher results with both detectors of the HPLC technique compared to the OPA/UV method (*p* > 0.05). Similar trends are depicted in Figure 3, where the CAC values of the standard fibers, expressed as adsorption percentage, are reported. The percentage deviations between different techniques applied to standard fibers can be observed in Figure S2, considering CAC expressed in both mg/g (A) and % (B).

Pectin exhibited CAC values above 80% with all the adopted techniques. Additionally, lignin exhibited high cholesterol adsorption percentages, with the highest value for OPA/UV. The interesting results obtained for pectin can be correlated to the high esterification degree of the tested commercial standard, apple pectin. It is known that pectin is a natural hydrocolloid present in plant cell walls, consisting of an acidic heteropolysaccharide, primarily structured around a linear backbone of D-galacturonic acid units. Based on the degree of esterification (DE), that is, the proportion of carboxyl groups esterified, pectin can be classified as high-methoxyl pectin (DE > 50%) or low-methoxyl pectin (DE < 50%). High DE can promote hydrophobic interactions between cholesterol and pectin, since the methoxyl groups endow pectin with high hydrophobicity to adsorb the nonpolar cholesterol molecule [3]. The cholesterol-lowering potential of high-methoxyl pectin extracted from Hylocereus polyrhizus peel was investigated by Zaid and colleagues, who concluded that the methoxyl content in the pectin acted as an affinity precursor of the pectin towards cholesterol due to its increased hydrophobicity [33]. Among the tested fibers in this work, lignin also exhibited good cholesterol adsorption capacity, considering the results expressed as mg/g (Table 5) and adsorption % (Figure 3). Other authors have reported that lignin, a highly branched polymeric structure composed of phenylpropane units with hydroxyl and carbonyl groups, is an important component of dietary fibers responsible for the adsorption of cholesterol and bile salts [34]. Regarding inulin, a significantly lower capacity for cholesterol adsorption was observed compared to pectin and lignin, as indicated by the results in Table 5 and Figure 3. Our results are consistent with those of a previous investigation on the effects of inulin and oligofructose on the in vivo adsorption and excretion of cholesterol [35]. The authors found that fructans had no effects on the in vivo adsorption and excretion of cholesterol. Lastly, for cellulose, the observed quite low values of cholesterol adsorption, considering both F and M cellulose, agree with those obtained by other authors. For example, Jin et al. [36] showed that cellulose could adsorb 5–10 mg cholesterol per gram of fiber, and Liu and Kong [37] reported that cellulose exhibited significantly lower cholesterol adsorption capacity (7.1 mg/g) than nano-fibrillated cellulose (8.5 mg/g). However, in the comparison of the results obtained in different investigations, it must be specified that several variables affect the absolute values of CAC, among which are biopolymer structural characteristics, fiber/cholesterol ratio, and environmental conditions.

### 3.4. Results of the Cholesterol Adsorption Capacity of Artichoke Leaf Fractions

The protocol developed for assessing cholesterol adsorption capacity has been tested on plant materials consisting of different fractions of artichoke leaves. It is widely known that the different parts of globe artichoke, *Cynara scolymus* L., are a promising source of dietary fiber. Artichoke leaves and stems are rich in total dietary fiber (>70 g/100 g dry matter), including soluble and insoluble fractions, as well as inulin [38]. While artichoke leaf extracts have been reported to improve lipid profiles [39], a systematic evaluation of leaf fractions for their cholesterol adsorption capacity remains lacking. Table 6 shows the results obtained by applying the developed procedure to four fractions of artichoke leaves. Their preparation was described in Section 2.2, while the chemical characterization has been reported in a recent article [21].

The results show that all investigated samples exhibited cholesterol adsorption capacity, with the outer part of artichoke leaves (A and B fractions) showing significantly higher values compared to the inner fractions (C and D). It is worth noting that this trend was confirmed by all the applied analytical techniques. However, while no significant differences were observed for each sample between the results obtained by the two detection systems of HPLC (*p* > 0.05), the OPA/UV technique gave higher and significant differences with respect to both the HPLC results (*p* < 0.05). In this regard, it is plausible to hypothesize that OPA spectrophotometry can overestimate the cholesterol adsorption capacity, potentially due to interferences. In fact, UV-Vis spectroscopy of plant materials is frequently challenged by significant spectral interference caused by the co-extraction of diverse pigments and secondary metabolites. The high content of chlorophylls, carotenoids, and phenolics creates overlapping adsorption bands in the UV and visible regions (200–800 nm), which can interfere with the signals of target compounds. These pigments act as interfering, non-selective signals that must be removed or mathematically corrected to achieve accurate quantification of target phytochemicals. For this reason, techniques like HPLC are often required to achieve higher selectivity.

**Figure 4:**
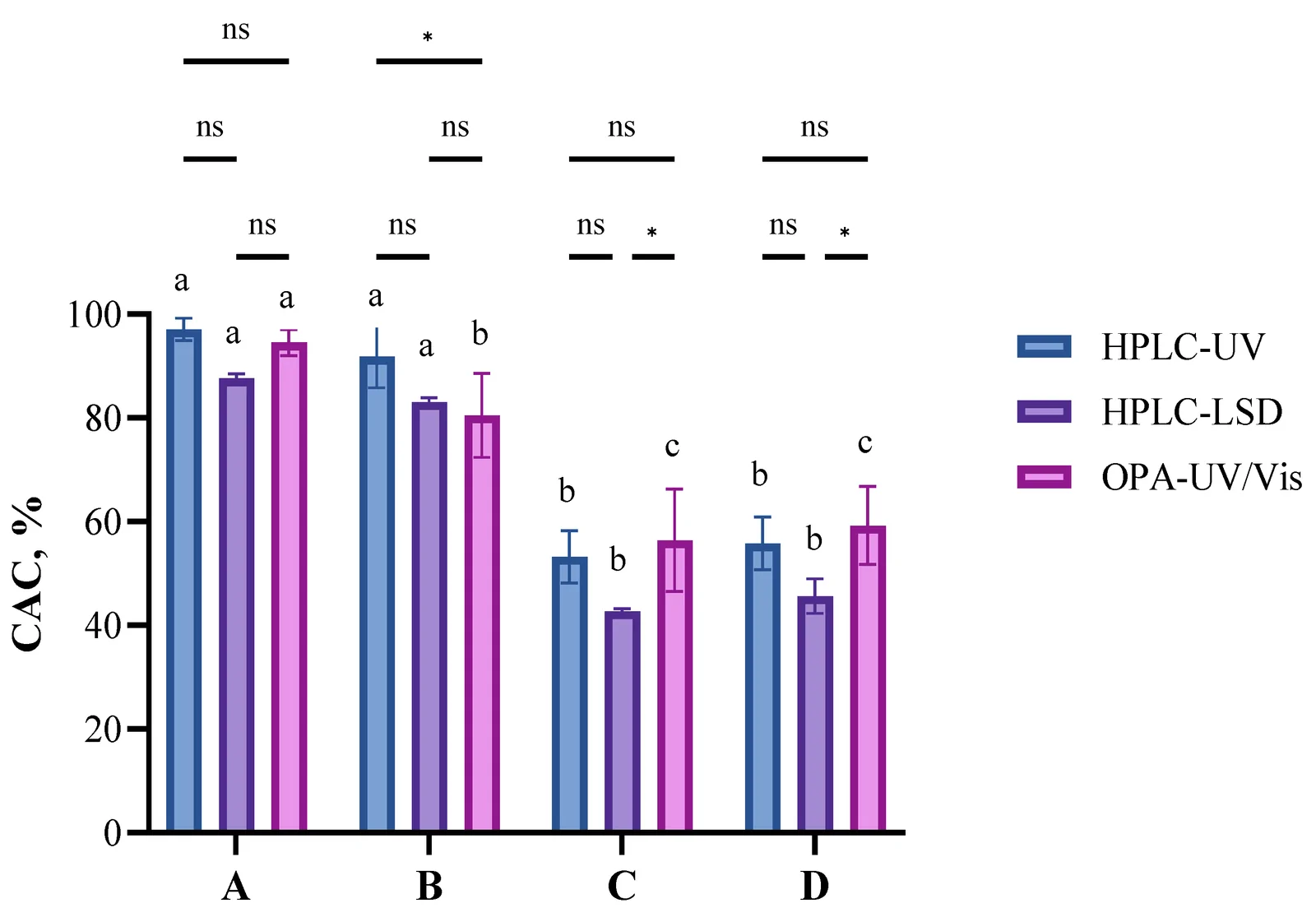
CAC of artichoke leaf fractions (A–D), expressed as adsorption percentage. Prism pairwise comparison shows differences between different analytical techniques for each compound: ns (*p* > 0.05, not significant), * (*p* ≤ 0.05). Different superscript letters above the bars indicate significantly different values (Tukey’s test, *p* ˂ 0.05) comparing the results of different compounds for each analytical technique.

The good results obtained for the investigated artichoke leaf samples, in particular for the A and B fractions, can also be observed in Figure 4, where the results of cholesterol adsorption capacity are expressed as adsorption percentage. Lower differences between the values measured by HPLC and OPA/UV for each sample have been measured, in fact statistically significant differences were observed only between HPLC/UV and OPA/UV in sample B, and between HPLC/LSD and OPA/UV in sample C and D. The percentage deviations between different techniques applied to artichoke fractions can be observed in Figure S3, considering CAC expressed both in mg/g (A) and in % (B).

As mentioned above, the comparison of the results obtained in this study with previous literature evidence is difficult, since, to our knowledge, there are no previous investigations on CAC in artichoke leaves. For this reason, Table 7 was prepared with evidence obtained in different plant materials with the objective of discussing, besides the CAC results, the analytical approaches followed by other authors.

First of all, Table 7 highlights that most published studies evaluated CAC under simplified experimental conditions, without simulating the sequential physiological events occurring during gastrointestinal digestion. Moreover, cholesterol quantification was mainly performed using OPA-based colorimetric assays with spectrophotometric detection at 550–560 nm. Only a limited number of investigations employed chromatographic techniques, despite their greater selectivity and analytical robustness.

The reported CAC values vary considerably depending on the plant material, fiber chemical composition, extraction process, structural modifications, as well as the analytical procedure adopted. For example, soluble fibers from apple peel and soybean hulls exhibited adsorption capacities ranging from approximately 6 to 11 mg/g [4,40], while modified dietary fibers obtained from black rice, bamboo residues, sweet potato, or quince showed markedly higher values, in some cases exceeding 50 mg/g or even 150 mg/g [8,43,45,46]. Particularly high adsorption capacities were reported for high-methoxyl pectin extracted from *Hylocereus polyrhizus* peel [33]. When compared with the literature data summarized in Table 7, the CAC values obtained for artichoke leaf fractions in the present study (approximately 9–20 mg/g by HPLC methods) can be considered competitive and within the range reported for several plant-derived fibers and agricultural by-products. In particular, the outer leaf fractions (samples A and B) exhibited adsorption capacities comparable to or higher than those reported for apple peel fibers, soy hull fibers, tea seed dietary fibers, potato dietary fibers, and *Ganoderma lucidum* dietary fibers [4,9,40,47].

It can be concluded that the experimental workflow proposed in this study, combining INFOGEST digestion and validated HPLC quantification, represents an important step toward the availability of a consistent approach for CAC determination and provides a robust framework for evaluating the cholesterol-binding potential of novel plant materials, including artichoke leaves.

## 4. Conclusions

This study presents a reliable analytical approach for evaluating the CAC of plant materials under simulated gastrointestinal conditions. The proposed protocol combines the INFOGEST static in vitro digestion model with cholesterol quantification using validated HPLC methods coupled with UV and LSD detection. Both chromatographic methods demonstrated satisfactory linearity, precision, accuracy, and sensitivity. The comparison with the conventional OPA spectrophotometric assay highlighted the higher selectivity and analytical reliability of the chromatographic approach. Although the OPA method is attractive because of its simplicity, it can overestimate CAC due to matrix interferences. These findings underline the importance of adopting chromatographic techniques for the accurate quantification of cholesterol, particularly for complex plant matrices.

Application of the developed procedure to commercial dietary fibers confirmed the expected differences among fiber types, with pectin and lignin exhibiting the highest CAC values. Furthermore, all investigated artichoke leaf fractions showed the ability to adsorb cholesterol, with the outer leaf fractions displaying significantly greater adsorption capacity than the inner woody fractions.

Further investigation could be worthwhile to assess matrix-matched recovery and determine whether the observed cholesterol reduction can be exclusively attributed to dietary fiber adsorption or whether potential losses may result from the experimental procedure.

The results of this study demonstrate that artichoke leaves, an abundant agricultural by-product, represent a promising source of functional ingredients with potential applications in foods and nutraceutical formulations aimed at supporting cholesterol management.

Overall, this work provides a methodologically consistent analytical framework that may improve the reliability and comparability of cholesterol adsorption studies while contributing new evidence on the health potential of artichoke leaf fractions. Future studies should investigate the relationship between fiber composition, physicochemical properties, and cholesterol adsorption mechanisms, and should further validate the physiological relevance of the proposed method through dynamic digestion models and in vivo investigations.

## Figures and Tables

**Table 1:** Basic structural unit and characteristics of the standard fibers.

	Structure	CAS Number	Aspect	Source	Impurity	Other
Cellulose Fibers (F cellulose)	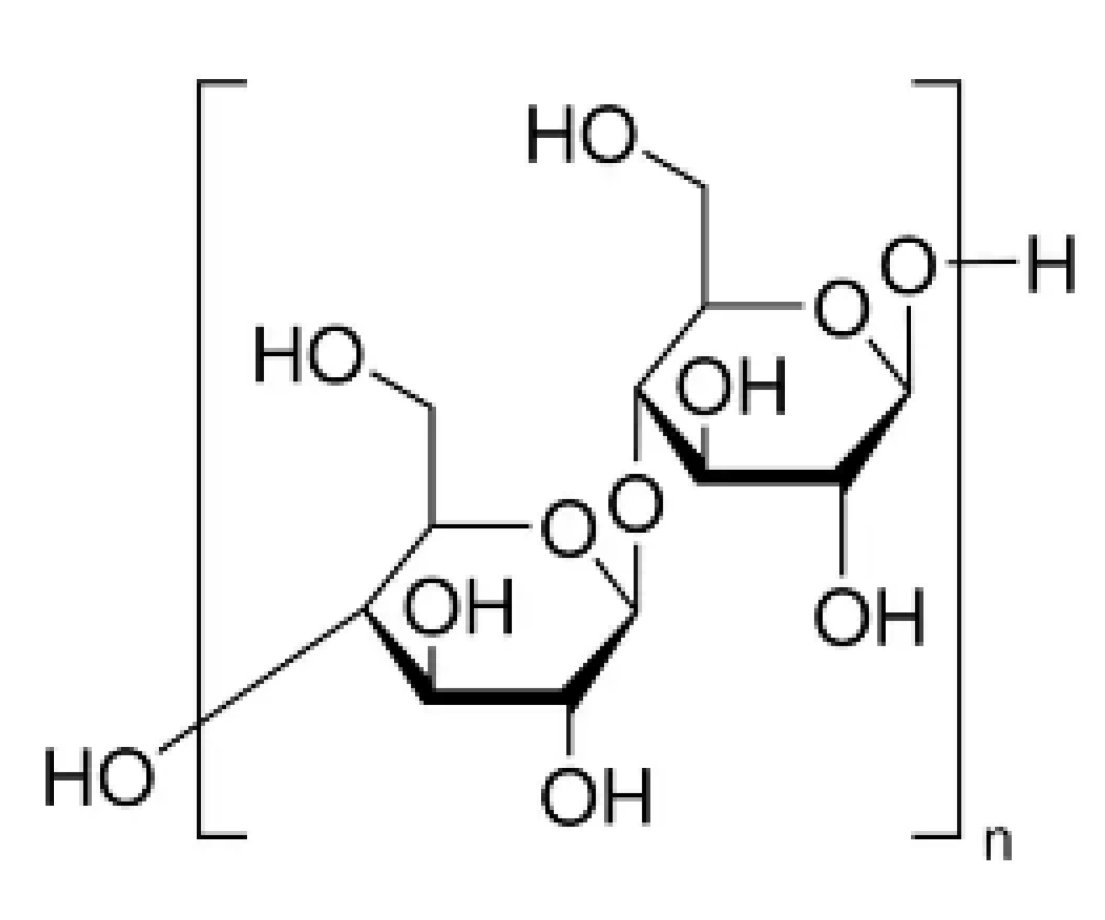	9004-34-6	White powder	Softwood tree pulp	Dark particles	Particle size (medium)
Cellulose Microcrystalline (M cellulose)	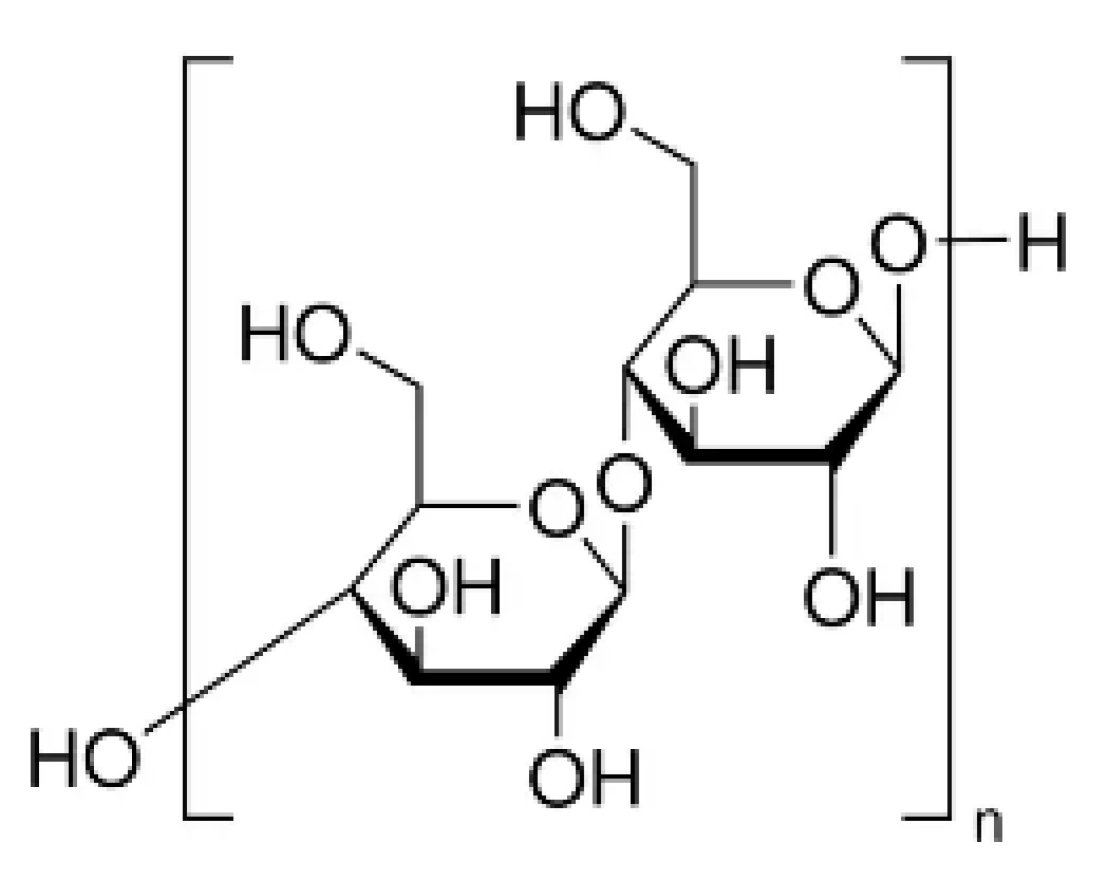	9004-34-6	White powder	Cotton linters		Particle size 20 μm
Inulin	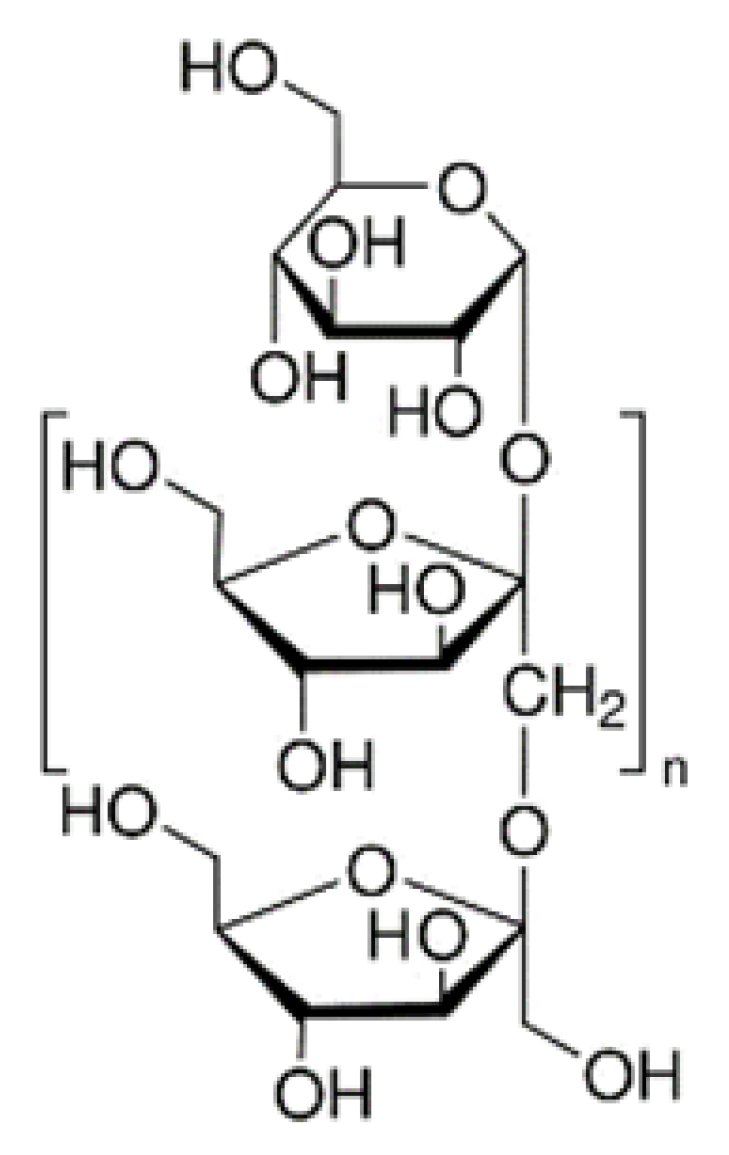	9005-80-5	White powder	Chicory	≤0.05% free glucose <15% water	
Lignin	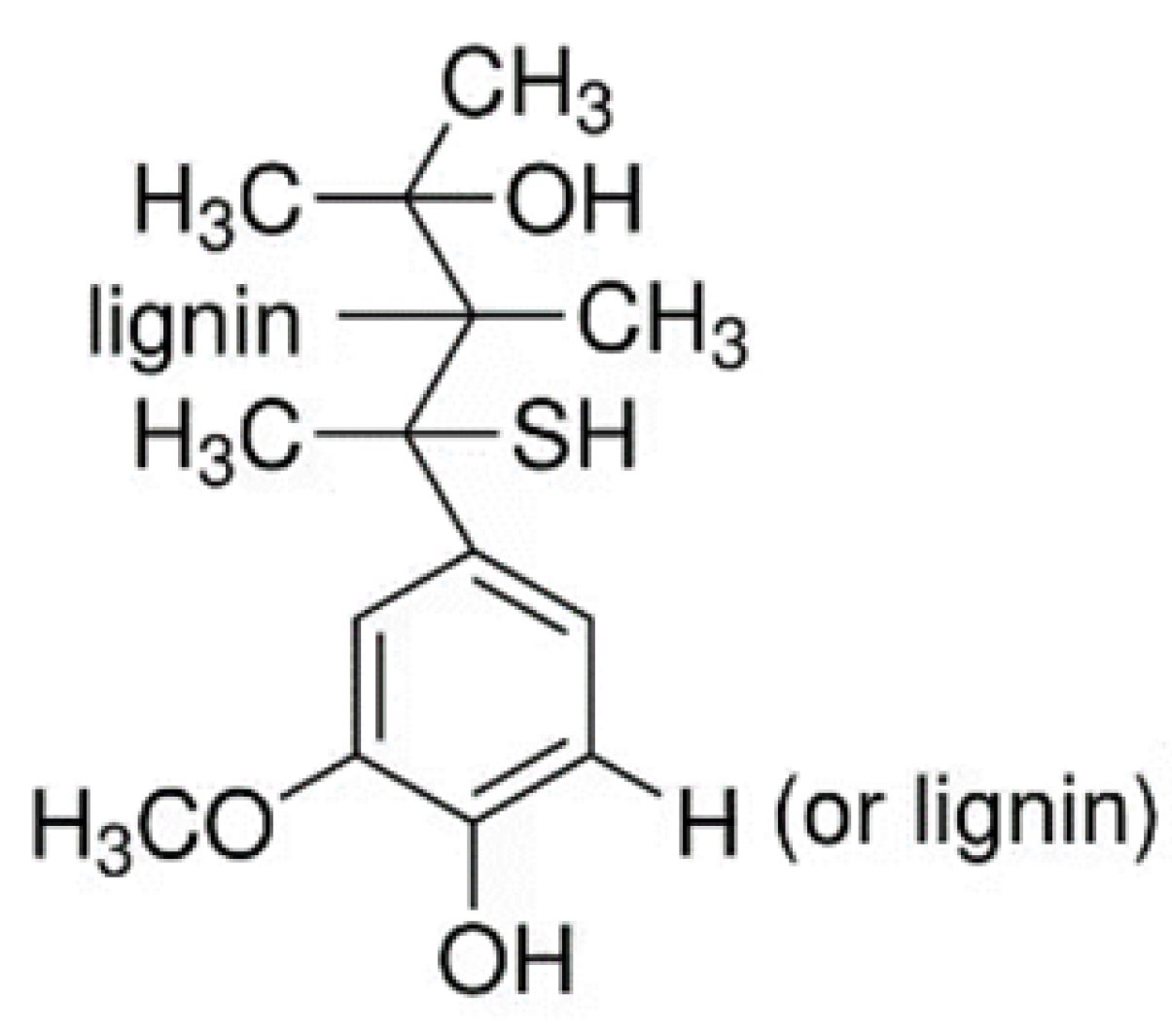	8068-05-1	Brown to black solid		4% sulfur	Average Mw ~10,000
Pectin	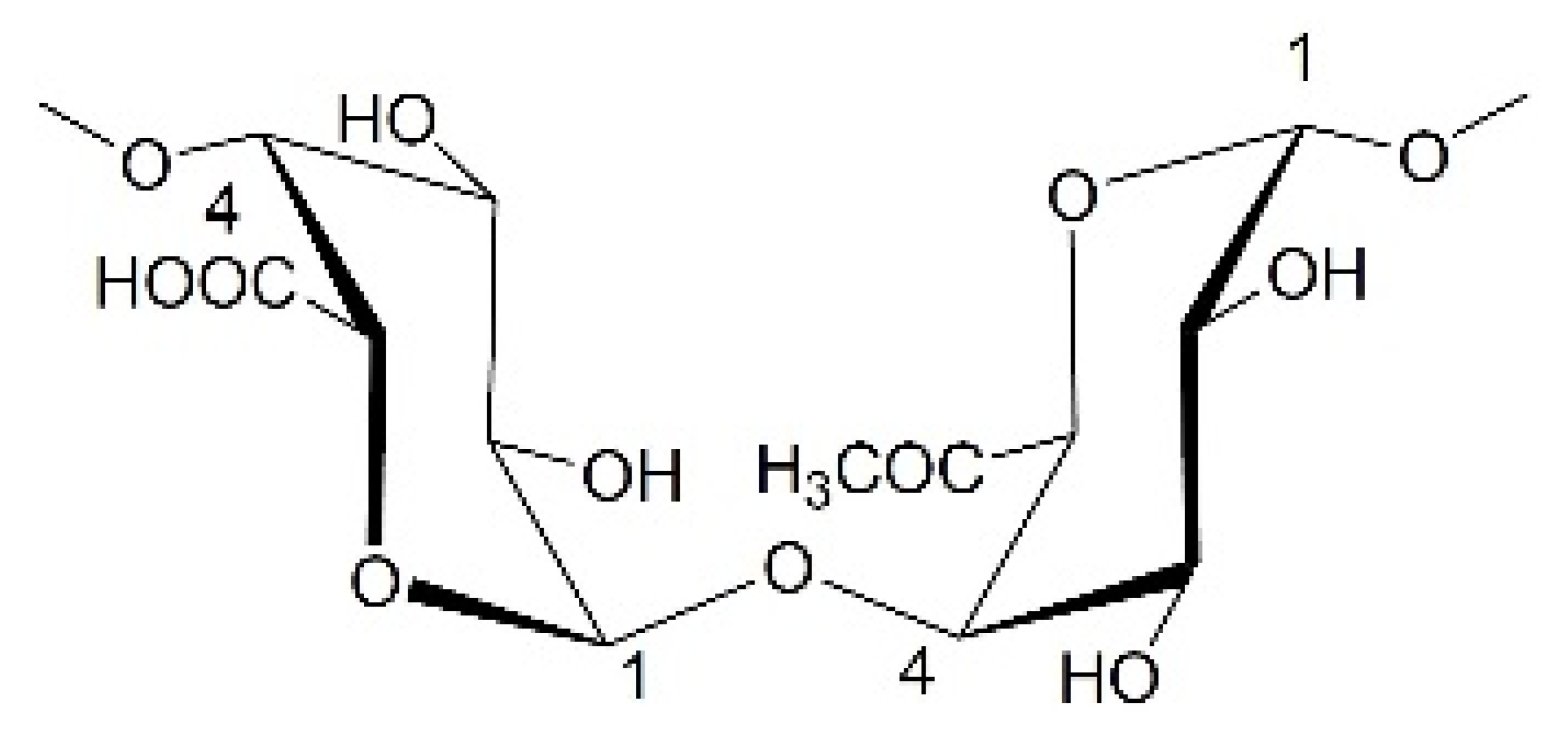	9000-69-5	White to light brown powder	Apple	≤10% water	50–75% esterification

**Table 2:** Artichoke leaf samples, particle size, and color parameters.

Artichoke Leaves		Particle Size	L*	a*	b*
Sample A	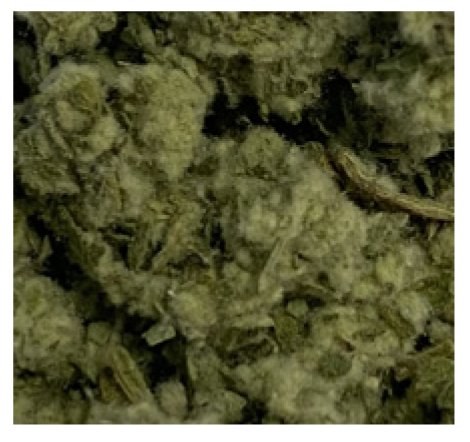	>1.18 mm	52.68 ± 0.01	−9.59 ± 0.01	22.16 ± 0.03
Sample B	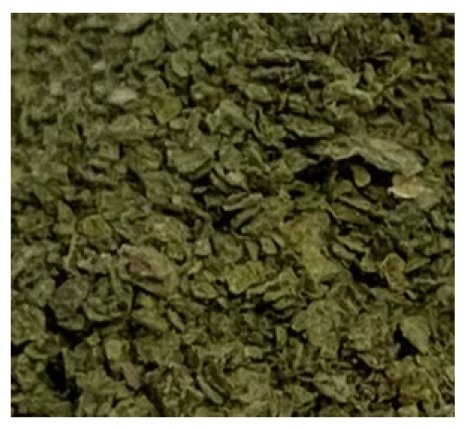	<1.18 mm	48.01 ± 0.01	−7.18 ± 0.01	20.07 ± 0.02
Sample C	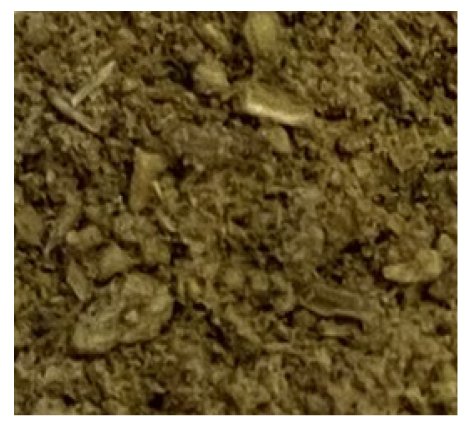	<1.18 mm	55.00 ± 0.01	0.21 ± 0.02	24.85 ± 0.02
Sample D	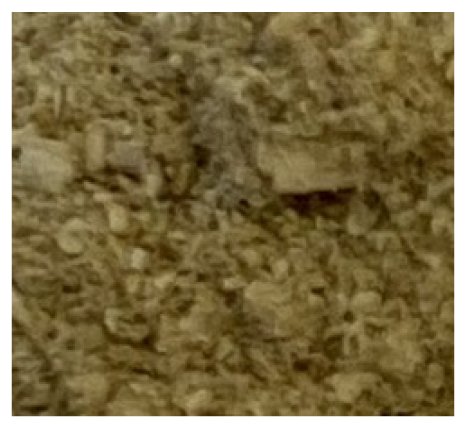	>1.18 mm	66.85 ± 0.02	−1.15 ± 0.02	24.39 ± 0.03

The images are representative photographs of the samples acquired using a smartphone camera.

**Table 3:** Regression equations, linearity ranges, correlation coefficient (R^2^) values, LOD and LOQ values at low- and high-cholesterol levels (LLV and HLV) using both chromatographic systems.

	Cholesterol	Regression Equation	Linearity Range (μg/mL)	R^2^	LOD (μg/mL)	LOQ (μg/mL)
HPLC/UV	LLV	y = 2812.25 (±55.46)x − 0.44 (±2.07)	1.75–73	0.9988	0.011	0.106
	HLV	y = 3954.39 (±406.65)x − 281.82 (±194.93)	146–750	0.9893		
HPLC/LSD	LLV	y = 1.58 (±0.11)x + 2.96 (±0.19)	3.5–146	0.9853	0.007	0.071
	HLV	y = 2.23 (±0.08)x + 3.61 (±0.04)	146–750	0.9988		

**Table 4:** Intra-day and inter-day precision and accuracy of cholesterol using both chromatographic systems.

	Theoretical Conc. (μg/mL)	Intra-Day Mean Concentration (mg/mL)			Intra-Day Mean Precision (RSD%)			Intra-Day Mean Accuracy (Recovery%)			Inter-Day Precision (RSD%)	Inter-Day Accuracy (Recovery%)
		Day 1	Day 2	Day 3	Day 1	Day 2	Day 3	Day 1	Day 2	Day 3	Day 1–3	Day 1–3
HPLC/UV	7.0	7.87	7.88	8.29	4.51	6.34	1.50	112.39	112.51	118.42	4.69	114.44
	219	219.95	224.09	229.96	1.26	1.25	1.49	100.44	102.23	105.01	2.26	102.59
HPLC/LSD	15	17.06	15.45	15.02	3.88	6.44	3.40	113.73	103.01	100.11	6.71	105.62
	292	303.02	309.39	308.47	0.42	0.79	0.93	103.78	105.96	105.64	1.17	105.12

**Table 5:** Cholesterol adsorption capacity of commercial fibers, expressed as mg/g.

Commercial Fiber	HPLC/UV	HPLC/LSD	OPA/UV
F Cellulose	3.72 ± 0.28 ^A,ab^	3.25 ± 0.35 ^A,ab^	-
M Cellulose	5.91 ± 0.41 ^A,a^	5.12 ± 0.17 ^A,a^	1.64 ± 0.32 ^B,a^
Inulin	2.70 ± 0.34 ^A,b^	2.48 ± 0.21 ^A,b^	5.31 ± 0.56 ^B,b^
Lignin	13.11 ± 2.35 ^A,c^	13.05 ± 2.11 ^A,c^	33.77 ± 1.99 ^B,c^
Pectin	17.32 ± 3.11 ^A,d^	18.53 ± 3.02 ^A,d^	28.66 ± 4.65 ^B,d^

Data are presented as mean and standard deviation (SD) (*n* = 3). Mean values followed by the same superscript lowercase letter within a column or the same capital letter within a row do not differ by Tukey’s test (*p* ˂ 0.05).

**Table 6:** Cholesterol adsorption capacity of artichoke leaf fractions, expressed as mg/g.

Artichoke Leaf Fractions	HPLC/UV	HPLC/LSD	OPA/UV
A	20.25 ± 0.63 ^A,a^	18.29 ± 0.25 ^A,a^	33.19 ± 5.85 ^B,a^
B	19.16 ± 1.09 ^A,a^	17.33 ± 0.24 ^A,a^	28.26 ± 4.02 ^B,b^
C	11.10 ± 1.49 ^A,b^	8.92 ± 0.13 ^A,b^	19.81 ± 4.91 ^B,c^
D	11.64 ± 1.49 ^A,bc^	9.52 ± 0.99 ^A,bc^	20.80 ± 3.76 ^B,cd^

Data are presented as mean and standard deviation (SD) (*n* = 3). Mean values followed by the same superscript lower-case letter within a column or the same capital letter within a row do not differ by Tukey’s test (*p* ˂ 0.05).

**Table 7:** CAC values of several plant materials, with the adopted experimental conditions.

Plant Material	Processing	Adsorption Conditions	Analysis	CAC, Maximum Values	Reference
Soluble fibers from apple peel, wheat bran, soybean-seed hull	-	pH 2.0 and 7.0	Colorimetry OPA-550 nm	Apple peel: pH 2.0, 10.75 ± 0.14 mg/g pH 7.0, 11.34 ± 0.15 mg/g	[40]
Fiber-rich extracts	-	Water-oil mixture	RP-HPLC in pellet phase	Pomegranate dietary fiber, >70%	[41]
Soluble fiber from soy hull	-	pH 2.0 and 7.0	Colorimetry OPA-550 nm	pH 2.0, 6.18 ± 0.27 mg/g pH 7.0, 7.62 ± 0.34 mg/g	[4]
*Hylocereuspolyrhizus* peel’s high-methoxylpectin	-	Tetrahydrofuran: water, 3:2	HPLC DAD 210 nm	Equilibrium uptake capacity 370.5 mg/g	[33]
Tea seed dietary fiber	Enzymatic and ultrasonic treatments	Simulated intestinal conditions	Colorimetry 550 nm	Ultrasonic treatment, pH 2.0, 12.71 mg/g pH 7.0, 8.67 mg/g	[9]
Plant-derived SDF	-	pH 2.0 and 7.0	Colorimetry OPA-550 nm	Konjac glucomannan, pH 7.0, 51.39 mg/g	[42]
Fiber from fermented black rice	Fermentation with *Neurosporacrassa*	pH 2.0 and 7.0	Colorimetry OPA-560 nm	Fermented black rice, pH 7.0, 59.22 ± 0.09 mg/g	[8]
Fiber from various agricultural waste	-	pH 7.0	Colorimetry OPA-550 nm	Bamboo fiber: 24.39 ± 0.44 mg/g	[43]
*Ganodermatocladium dietary fiber*	-	Simulated intestinal conditions	Spectrophotometry	SDF, 11.2 mg/g	[9]
Black rice bran IDF	Steam explosion, extrusion, high-pressure steaming	pH 2.0 and 7.0	Colorimetry OPA-550 nm	pH 7.0 (22.02–28.18 mg/g) pH 2.0 (7.73–11.27 mg/g)	[44]
Quince (*Cydoniaoblonga* Miller) fiber fractions	Acid, alkaline, enzymatic extraction	pH 7.0	Colorimetry OPA-550 nm	Acid extraction: SDF (155.74 mg/g) IDF (44.65 mg/g)	[45]
Sweet potato fibers	Enzymatic hydrolysis, ball milling, extrusion	pH 2.0 and 7.0	Colorimetry OPA-550 nm	After removing non-fiber components, extrusion treatment (26.54 mg/g at 140 °C)	[46]
Potato (*Solanumtuberosum*) dietary fiber	High hydrostatic pressure and cellulase	pH 2.0 and 7.0	Colorimetry OPA-550 nm	pH 7.0, HHP–assisted cellulase-modified DF, 7.13 mg/g	[47]

## Data Availability

The original contributions presented in this study are included in the article/Supplementary Material. Further inquiries can be directed to the corresponding author.

## Supplementary Materials

The following supporting information can be downloaded at: https://www.mdpi.com/article/10.3390/foods15173099/s1. Chromatographic profiles with both UV–Vis (left column) and LSD (right column) of the cholesterol adsorption capacity of standard fibers: (A) F Cellulose; (B) M Cellulose; (C) Inulin; (D) Lignin; (E) Pectin; Figure S2. Percentage deviations between different techniques applied to standard fibers; Figure S3. Percentage deviations between different techniques applied to artichoke leaf samples.
