# Supplementary material for: Reliable Analytical Approach for the Quantification of the Cholesterol Adsorption Capacity of Artichoke Leaves
Source: Foods. 2026 Aug 31;15(17):3099. doi: 10.3390/foods15173099 (PMC13564901; doi:10.3390/foods15173099)
Supplement: Supplementary file 1 [file foods-15-03099-s001.zip › foods-4489580-supplementary.pdf]

Article

Reliable analytical approach for the quantification of the cholesterol adsorption capacity of artichoke leaves

Shahd Ali, Federica Ianni *, Lina Cossignani

Department of Pharmaceutical Sciences, University of Perugia, via S. Costanzo, Perugia, 06126, Italy; shahd.ali@dottorandi.unipg.it (S.A.); lina.cossignani@unipg.it (L.C.)
* Correspondence: federica.ianni@unipg.it

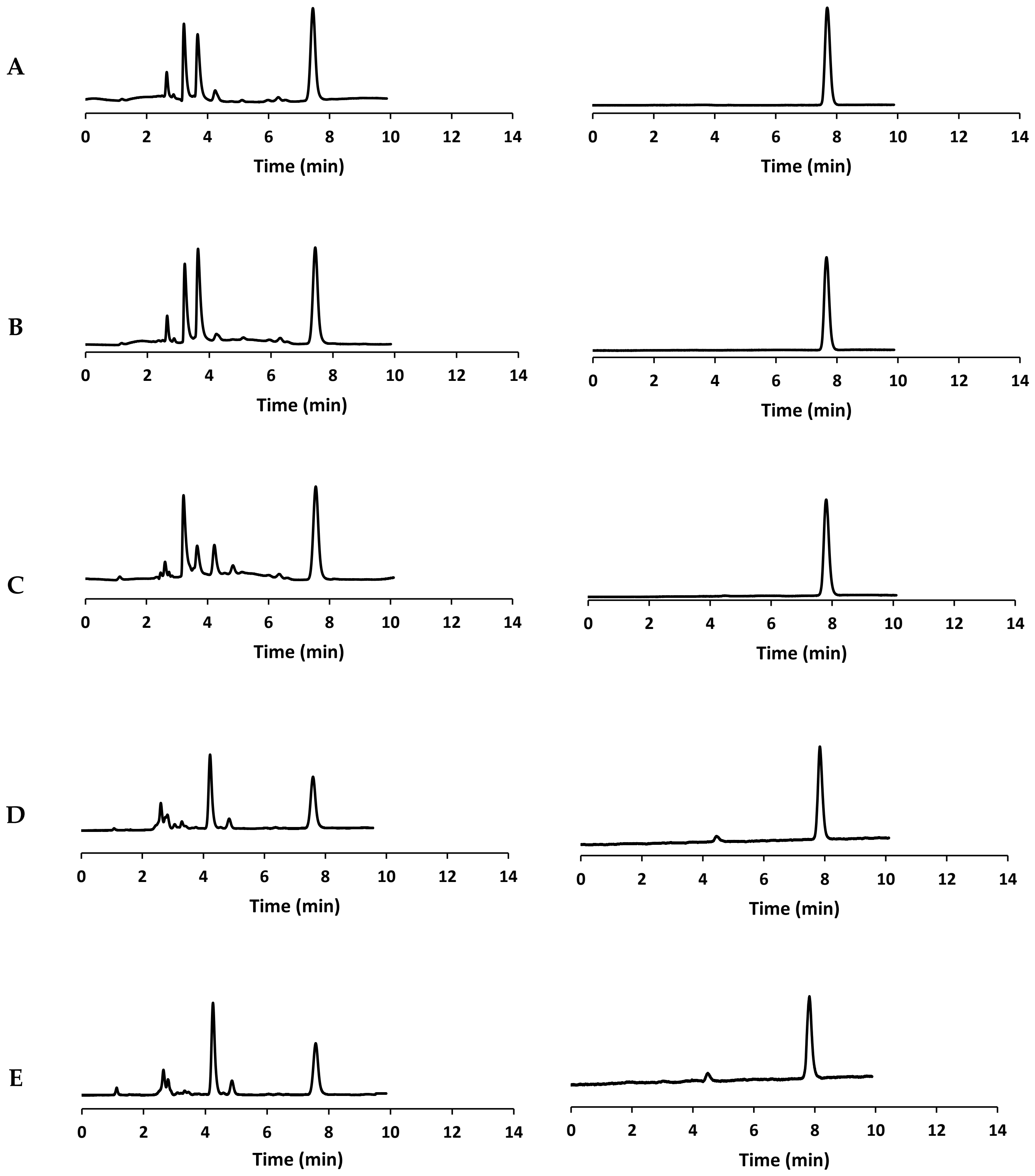

Figure S1. Chromatographic profiles with both UV–Vis (left column) and LSD (right column). of the cholesterol adsorption capacity of standard fibers: (A) F Cellulose; (B) M Cellulose; (C) Inulin; (D) Lignin; (E) Pectin.

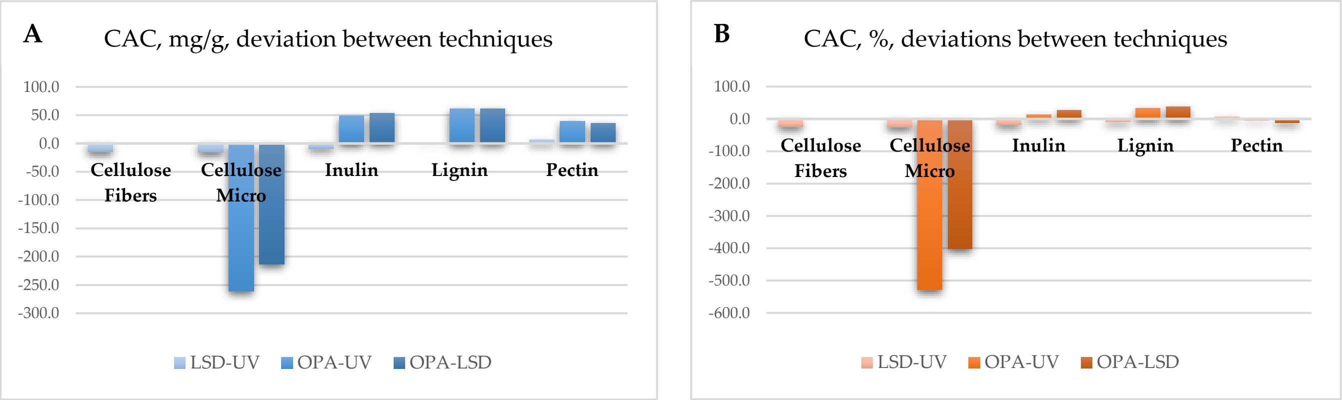

Figure S2. Percentage deviations between different techniques applied to standard fibers.

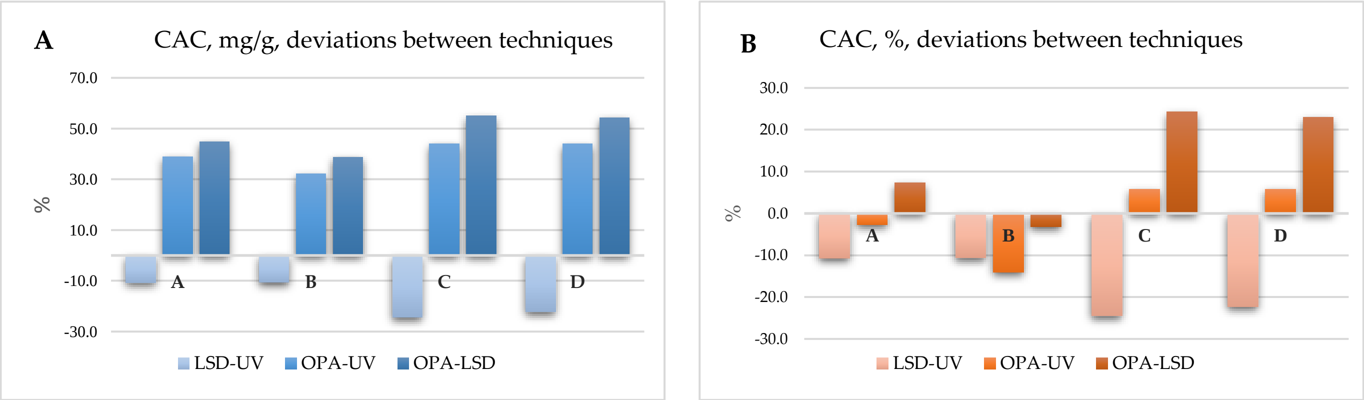

Figure S3. Percentage deviations between different techniques applied to artichoke leaf samples.
